# How Shall We Feed the Baby?*Read before the Washington Obstetrical and Gynecological Society, May 3, 1895.

**Published:** 1896-09

**Authors:** John T. Winter

**Affiliations:** Professor of Theory and Practice of Medicine, National University, Washington, D. C.


### Selections and Abstracts.

### HOW SHALL WE FEED THE BABY?*

By JOHN T. WINTER, M. D.

Professor of Theory and Practice of Medicine, National University, Washington, D. C.

It should be the pleasure of every mother to nourish her babe from her own breast, and, to insure a proper understanding of what is expected of her, it is the duty of the attending physician to instruct her in some of the details of motherhood with which she is so soon to become familiar. It is also his duty to see that the child is placed in a proper position at the mother's breast within the first half-hour after its birth, or as soon thereafter as the mother has recovered somewhat from the fatigue of labor. I do not claim that the child will get milk from its mother's breast at this early period, but it will get, in a majority of instances, a small quantity of colostrum, which not only affords some nourishment to the infant, but, because of its laxative properties, clears out its intestinal canal and has a beneficial action on the mother, as it not only hurries the secretion of milk, but causes uterine contraction. It also serves another good purpose in affording peace of mind to the attending physician, as he will know by this little attention on his part that the child can nurse, and that there is proper uterine contraction and therefore no danger of hemorrhage.

But this is not always the whole question. Natural lactation is undoubtedly the method which ought to be preferred, if practicable, but to this general rule there are many exceptions. A woman to be a good nurse should enjoy perfect health, should be rather spare than fat, and should be cheerful, good-natured, active, careful, and above all, temperate. This will apply as well to a wet-nurse as to the mother herself.

The manner in which the menstrual function is performed may, to a certain extent, be regarded as an indication of the

*Read before the Washington Obstetrical and Gynecological Society, May 3, 1895.

manner in which milk will be secreted. If menstruation be irregular and scanty there is fear that the lacteal secretion will be badly accomplished, and when the menses are habitually too abundant, nursing fitness is also improbable. But when this function has always been performed normally, when the woman is regular in time and quantity, the presumption is in favor of her making a good nurse. It is necessary, therefore, for the physician to inform himself in all these particulars, so that he may be able to anticipate and direct the mother in the feeding of her child, and how to avoid some of the errors they seem so ready to hunt up and adopt. Some of these errors are frequently begun within the first hour of the child's life. It will be found in many instances that the little stranger has scarcely finished his first bath when, if you are not on the lookout to prevent, the nurse will begin her part of the mischievous work by dosing it with a little sugar and water, catnip or chamomile tea, or some other decoction of a similar character, which she many times carries with her to the patient's house; and while you may prevent this dosing as long as you remain in the house, you will find in the majority of instances, even when she denies it, that before your next visit she has gotten in her work.

I can readily understand that the young physician can ill afford to brook the opposition of the old nurse, who perhaps was nursing before he was born, and may have nursed in her infancy the very woman he is now called to attend; but he can much less afford to curry favor by conniving at her pernicious practices. The ingestion of the vile stuff with which the infant is so frequently dosed during the first day or two of its life is the first step toward the formation of that bugbear of old women, the red-gum, an erythematous lichen—lichen strophulus—an affection brought on by the very means adopted to guard against it; and unless the physician has a sufficiently strong hold on the confidence of the family to enable him to correct at once this first piece of ignorance and meddlesomeness, he will find that it is but the beginning of a course of unnatural feeding which will, in many instances, bring death to the child.

In nursing or feeding an infant, regularity in point of time should be observed. During the first week every two or three hours in daytime will not be too often, and at night only if mother's breast needs to be unloaded. By the end of the second week, as a rule, when the child is strong and healthy, to be nursed or fed once in three hours during the day and up to the mother's bedtime, and then not at all for six or seven hours, when both mother and child should sleep, will be found amply sufficient. The child should never be given the breast or bottle simply to keep it quiet, but should be taught regular habits in this direction. You will meet with opposition from the nurse, who will be supported in her opposition by all the old women in the neighborhood, and you will be informed, perhaps, as I have been many times, that you have never had a baby and that women only have any knowledge on this subject. Many of my patients have, however, adopted this method, and have thanked me over and over again for the comfort they have with their children.

A nursing mother in good health will generally grow fat during the first few months of lactation, but this plumpness will gradually be lost if nursing is continued too long, or if the child is allowed to nurse at night, especially if it is allowed to lie at the breast and nurse when it pleases. Under such circumstances it will not be long before the mother's milk will cease to nourish the child, or if it does so it will be at the expense of the mother's health. I have seen some terrible examples of this kind—young mothers scarcely able to get out of bed in the morning, and who claim to be more tired, if possible, than when they went to bed the evening before. Among the early symptoms of failure from this cause are heavy dragging sensations in the back and loins, and directly between the scapulæ when the child is at the breast, and a peculiar feeling of sinking and emptiness at the pit of the stomach and over the whole abdomen for hours afterward. I have a patient of this kind under my care at the present time.

Under such a condition of affairs the question of artificially stimulating the lacteal secretion would naturally follow; but, as I have already put myself on record on this subject, I will only say that while temporarily stimulating this secretion during the illness of a child, for example, is frequently proper and should be done, the continued use of beer, ale or other preparations of this kind will be found to keep up or increase the quantity only at the expense of quality, and it will soon be found that the mother is not only being seriously injured, but that the child is not being nourished.

While there are many honorable women in the land who do suckle their own children, and many mothers who would be delighted to do so, but can not or should not, as they are many times the subject of some physical or mental disturbance which unfits them in every particular for this pleasant duty, there are others who will not nurse their children, not for any particular reason, only that they do not care to do so. When, therefore, the mother can not or will not nurse her child, and where a suitable wet-nurse can not be secured, some other method will have to be adopted, and the child has a right to the nearest and best substitute for mother's milk with which we can supply it, and that substitute is usually found in the milk, or some of the products of the milk of the cow. The milk of the goat and ass is said to more closely resemble woman's milk than that from any other animal, but these animals are not kept in sufficiently large numbers to supply the milk required in the artificial feeding of children. The milk of the cow is, therefore, the nearest practical substitute for that of the mother.

Human milk has a specific gravity of 1031 and has a persistently alkaline reaction, and shows, when placed under a microscope, a number of minute, equal-sized fat globules; it coagulates into flaky curds, and is quite readily attacked by the digestive ferments of the child's stomach and intestines; while cow's milk has an average specific gravity of 1029, and unless perfectly fresh and from pasture-fed cows, has a slightly acid reaction, contains less fat and sugar, but is richer in casein and coagulates into a very firm curd, and is therefore much harder for the child to digest. This difference is readily tested by adding rennet to the two fluids. Under its use the milk of the cow coagulates into large, firm masses, while with human milk a large, loose curd is formed.

The chemical and physical properties of cow's milk can be altered by various methods of preparation, so as to make it approach more closely to mother's milk; but, unless this is done, it will be found, in the majority of instances, to prove but a poor substitute for the natural food. The majority of rules given on this subject are too complicated for practical use, but by simply diluting cow's milk with boiled water, lime-water, barley-water, rice-water, or oatmeal, the firmness of the curd may be prevented, and by adding sugar-of-milk we have a very good substitute for mother's milk. Cream should also be added, as cow's milk is not so rich in this particular to commence with, and the addition of the water further reduces its strength. The specific gravity of cow's milk will average 1029, but, unfortunately, there is no instrument which will indicate its purity or quality. It is claimed that while lactose, salts, and casein increase the specific gravity of milk, fat and cream, being lighter than water, decrease its specific gravity; therefore, if cream be removed the specific gravity will be increased, and in order to bring it back to the standard, 1029, water may be added—that is, cream may be removed from, and water added to, the same milk, and the specific gravity will remain unchanged. A rough test can be made, however, which will in a measure determine the quality of the milk. A tall, graduated glass cylinder should be filled with the milk to be tested, which, after standing ten or twelve hours, should, if pure, throw up at least ten per cent. of cream and ought not to deposit any solid matter.

The question as to whether milk should be given fresh or boiled is one that should receive some attention. There is no question but that the heat of sterilization robs the nuclein of the milk of its vital properties to some extent, but where we are dependent on the dealer who receives his supply of milk from the country and has it in his dairy a number of hours before it is delivered to his customers, it should be either sterilized, pasteurized, or boiled; but where it can be had fresh and free from bacteria it can be used in the raw state and can be warmed sufficiently by the water with which it is diluted, and it is thought in this way to be more nourishing and more easily digested.

I do not think it is wise to habitually peptonize milk for healthy children, as their digestive organs, not being called on for full duty, are, I fear, weakened; and the careless manner in which the sterilizing of milk is frequently done makes it many times rather harmful than beneficial, as it makes the coagula of casein in cow's milk much harder to digest.

I never advise the use of milk from a single cow, in the city, unless the cow is owned and fed by the parents or friends of the child, but advise that the milk be taken from an honest dairyman and drawn from the large can he usually has in his wagon, feeling that the milk from a good herd of cows is less likely to be injurious than if taken from any one cow of the same herd; and preferably from the can, unless each family supplies and cleans its own bottles, as those supplied by the dealer are frequently not any cleaner than they ought to be.

I have long objected to the use of condensed milk in my practice, but have been compelled to submit to its use over and over again, and have seen children who seemed to do as well on it as on any other artificial food, especially during the summer months when the milk they were depending on would be sour many times when it was received. Several years ago, as you will no doubt all remember, some of the clergy of our city succeeded in limiting the dairymen to one delivery of milk on Sunday, and in stopping the ice dealers from supplying ice on Sunday to their customers at all, insisting that the consumer should lay in a sufficiently large supply on Saturday to last until Monday—not knowing or caring, perhaps, that their action would deprive many poor persons of the use of this luxury, and would compel them to find a substitute for the cow's milk on which their children were living, which many of them have done, and are now using condensed milk, not only for their children, but in their coffee.

My objection to the use of condensed milk is that while the infant will fatten on its use, it is usually found to be pale and flabby, and does not, many times, have strength enough to carry it through childhood. By the addition of cream and the water from barley or rice, or a thin oatmeal gruel, we can improve it in this particular.

It is hardly necessary for me to say much or anything about the various patented preparations, each more marvelous than the other, as I very much prefer milk or some of its products for the artificial feeding of children. Those, however, which contain a smaller per cent. of fat than does a healthy mother's milk are more prone to cause disturbances of a developmental character, observed most frequently in the osseous system; hence rachitis, which is a comparatively unknown disease in rural districts where children enjoy the hygienic requirements of pure air, sunlight, and wholesome food, is found to be not uncommon among the crowded and badly-fed families in city tenement houses where anti-hygienic conditions prevail.

It is claimed that in Japan there is no artificial feeding of children, and that milk, being an animal product, is excluded as a food, and the Japanese mothers are, therefore, compelled to suckle their babies themselves, which they do until their children are five or six years old, the mothers not menstruating during the first two years of lactation. The principal food of the mothers, and of the children after they are old enough, besides the everlasting rice, is composed of fats and oil of fish, seaweeds, and other products of the sea. No wine or beer ever enters into her diet, and the great reward Japan is said to reap from this care is the entire absence of rachitis. It is also claimed that the total abstinence of the Japanese from drinking cold water has made typhoid fever unknown in that country, where the water is said to be highly polluted, water being used by them for drinking only when it is boiled with tea.

The great principle at the foundation of all successful feeding of either adult or infant is that the supply of food be proportioned to the ability of the individual to digest. During infancy the stomach appears to be peculiarly liable to disturbances of its function, and these disturbing agents seem to be multiplied during the heat of summer—less so, of course, for those at the breast, but still errors in diet on the part of the mother or disturbances of her nervous system will cause changes in the milk, and thus cause gastric disturbance. That this is correct can easily be proven, simply by giving the mother a dose of castor oil. In young children stomach digestion is but imperfectly performed at best and is of less importance than intestinal digestion. The stomach is more of a receptacle, into which the milk is received for coagulation, than a digestive organ, and the younger the child the less the stomach has to do, the milk simply being coagulated and passed into the intestines undigested. Even the digestion of the constituent of milk, milk-sugar, is now thought to be accomplished by a ferment found in the mucus of the small intestines.

Starr, in his new work, "An American Text-book on the Diseases of Children," says: "The experiments in this direction convince us that the digestion of milk by the infant is nearly if not altogether accomplished in the small intestines, and explains why indigestion in the infant induces diarrhœa."

Excessive feeding is quite frequently the cause of intestinal indigestion, especially in artificially-fed children, for the reason that the food is literally poured into the stomach and intestines. The mother will many times give her child the bottle when she would not stop to nurse it. The overloading of the stomach in this way causes more work than the stomach is capable of performing; the food must, therefore, either be vomited up or passed on into the intestinal canal without being properly prepared, and thus cause intestinal indigestion.

I was called, a couple of years ago, to a case of this kind occurring in the family of a physician. The child, six months old, had just been brought home from the country, and was suffering with a terribly offensive and curdy diarrhœa, which had existed already for several weeks. The child was very much emaciated, very fretful, whining uneasily almost all the time, and would rarely ever sleep more than ten or fifteen minutes at a time, unless it was given large doses of paregoric or bromide of potassium. The father sent for me, as he had given all the medicines and all the foods he had ever heard of as being good for such a condition of affairs. He kept an Alderney cow in his stable, and, in addition to the pure milk which he was using, he was adding a tablespoonful of Mellin's food to each nursing-bottleful of milk, which would be offered to the child every time it cried, frequently several times in a single hour, and the bottle refilled every time it became empty, without regard to time. The father became very indignant when I suggested that he was giving the child too much food; that the indigestion which was causing the diarrhœa would take care of itself if he would stop the Mellin's food and reduce the milk one-half by adding rice-water or simply boiled water, which he refused flatly to do; but after considerable talk he was induced to try my plan for a few days, and was rewarded by having his child restored to health. Many times in the last few years I have entirely stopped the use of milk for a day or two at a time when children were suffering from gastro-intestinal indigestion. To such children I many times give a teaspoonful or two of beef juice several times a day, and the water from toast bread, rice, or pearl barley; and while I have allowed all the water they wanted, I have returned but slowly to their regular, every-day supply of milk. And this is, perhaps, as good a place as any to call attention to the fact that a sufficient supply of water is frequently not given to young children.

Great care should be given not only to the preparation of the food, but to see that the bottle and nipple are kept thoroughly clean. The graduated nursing-bottle is the best, as it is smooth inside and has no tube to be kept clean. The black rubber nipple should be used, and turned inside out when washed. Many persons having charge of bottle-fed children are exceedingly careless in all these particulars. They may be careful enough in the preparation of the milk in the first place, but after filling a bottle they will allow a child to nurse from it several times before it is all gone, and, without cleansing the bottle, will refill it again and again, or will many times add fresh milk to what has been left over from a previous filling; and they are quite as careless with the nipple. If you have any doubts on this subject, turn a rubber nipple inside out some warm day this summer and convince yourself.

A case occurred in my practice a few years ago in which a little more than the ordinary degree of carelessness was exhibited. A young woman volunteered to take care of her sister's children for part of a day, and in going to her house and finding the baby, nine months old, crying, picked up a bottle that had been filled the day before with raw milk—which was now quite sour, the curd having separated from the whey—and shaking it up, gave it to the child, repeating this several times until the bottle was empty. It is hardly necessary to say that in about twenty hours the child was dead.

During the first week or two after birth the child should be fed every two or three hours, and will require about one ounce at each feeding, about half a pint a day—good milk, two drachms; boiled water, rice-water, or barley-water, six drachms.

By the second week, and up to about the sixth week, it will require about one pint a day, and unless the milk is first-class, it will be well to add sugar-of-milk and cream—sugar-of-milk, four grains; cream, one drachm; milk, four drachms; water, one and a half ounces.

At about the third to the sixth month it will take nearly four ounces at each meal, nearly a quart a day—sugar-of-milk, one scruple; cream, two drachms; milk, one ounce; water, two ounces.

From this time on about the same proportions should be maintained, the quantity being gradually increased until three pints—perhaps a little more—are taken in twenty-four hours, the quantity of water being gradually reduced, or a thin oatmeal gruel can be substituted for the rice or barley-water. A small pinch of salt can occasionally be added. This gruel can be gradually thickened, so that by the time the child is ten or twelve months old it can have, say, two meals a day of oatmeal gruel and three of pure cow's milk.

It is better for the child up to this time to keep it from the table and not allow friends or strangers to give it candy, cakes or other food, but from this time on, potatoes and other suitable articles of diet can be added.—*The American Journal of Obstetrics.*

